# Occupational Stress, Work-Family Conflict and Depressive Symptoms among Chinese Bank Employees: The Role of Psychological Capital

**DOI:** 10.3390/ijerph13010134

**Published:** 2016-01-16

**Authors:** Dan Kan, Xiaosong Yu

**Affiliations:** 1 School of Public Health, Harbin Medical University, Harbin 150081, Heilongjiang, China; kandan30@163.com; 2 Department of General Medicine, The First Affiliated Hospital of China Medical University, Shenyang 110001, Liaoning, China

**Keywords:** occupational stress, work-family conflict, psychological capital, depressive symptoms, bank employees

## Abstract

Although depression is a major problem affecting the physical and mental health of the occupational population worldwide, little research is available among bank employees. The purpose of the study was to examine the effects of occupational stress and work-family conflict on depressive symptoms and the mediating role of psychological capital (PsyCap). A cross-sectional study was performed from May to June in 2013 in Liaoning province, China. The effort-reward imbalance (ERB) scale, the work-family conflict scale, the PsyCap questionnaire and the Center for Epidemiologic Studies Depression scale were completed by 1546 employees in state-owned banks. A total of 1239 effective respondents (467 men and 772 women) became our subjects. Hierarchical regression analysis was carried out to explore the effects of extrinsic effort, reward, overcommitment, work-family conflict, and PsyCap on depressive symptoms. The mediating role of PsyCap was examined using Preacher and Hayes’ asymptotic and resampling strategies. The mean score of depressive symptoms was 18.4 (SD = 7.6) among the Chinese bank employees. Extrinsic effort, overcommitment and work-family conflict were positively associated with depressive symptoms. Reward and PsyCap were negatively associated with depressive symptoms. The significant mediating roles of PsyCap in the associations of extrinsic effort (a*b = 0.046, BCa 95% CI: 0.029, 0.066) and reward (a*b = −0.047, BCa 95% CI: −0.065, −0.030) with depressive symptoms were revealed. There is a high level of depressive symptoms among Chinese bank employees. PsyCap partially mediates the effects of extrinsic effort and reward on depressive symptoms. Investing in PsyCap may provide new approaches to improve mental health among Chinese bank employees.

## 1. Introduction

Depression has become one of the most common medical problems worldwide. In workplaces, depression has an important influence on the quality of life of workers, and can result in direct economic costs by reducing productivity [1], because depressive symptoms have an impact on their decision-making and ability to get along with others [2]. In addition, depressive symptoms are associated with unemployment and loss of family income [3]. World Health Organization (WHO) estimates that depression will become the second-leading cause of disability worldwide by 2020 [4].

Previous studies in various populations suggest that social anxiety, work-related stress, and stressful life events may contribute to depression [5,6,7]. Various occupational stressors have been identified as potential risk factors for depressive symptoms [8]. The level of occupational stress can be assessed using the effort-reward imbalance (ERI) model, which focuses on reciprocity of extrinsic effort, reward, and intrinsic effort (overcommitment) [9]. Kinman and Jones reported that high effort and low reward within the ERI model significantly predicted negative outcomes, including psychological distress, physical symptoms, and low job satisfaction among UK university employees [10]. In China, the effect of occupational stress assessed by ERI model on depressive symptoms has been confirmed among various occupational populations, such as nurses, physicians, male correctional officers, university teachers, and underground coal miners [11,12,13,14,15].

There are two important focal domains of adult life: work and family. However, the role expectations of work and family are not always compatible, which can create conflicts between work and family. Netemeyer *et al.* defined work interfering family conflict (WIF) as “a form of interrole conflict in which the general demands of, time devoted to and strain created by the job interfere with performing family-related responsibilities” [16]. Work-family conflict has positive effects on workers’ turnover, burnout and depressive symptoms [17,18,19]. In addition, the effects of work-family conflict on burnout and depressive symptoms have been confirmed across Chinese occupational populations including doctors, nurses, and underground coal miners [15,20,21,22].

According to the results of previous studies, occupational stress and work-family conflict not only exert a direct effect but also have an indirect effect on depressive symptoms through inhibiting specific psychological responses [12,14,22]. Recently, many studies have attempted to identify psychological capital (PsyCap) as a positive resource for combating mental health problems across occupational groups [12,14,20,21,22]. PsyCap is advocated for “the study and application of positively oriented human resource strengths and psychological capacities” [23]. PsyCap is a high-order construct that is comprised of four state-like psychological resources: self-efficacy, hope, optimism, and resilience, which can be measured and developed [23,24]. Specifically, when employees possess high PsyCap, they are confident to complete their work tasks, are more hopeful under negative working situations, expect good things to happen, and quickly “bounce back” after setbacks [14]. PsyCap has been validated and put into application gradually in various fields, including businesses, medicine, and education [14,23,24,25,26]. Previous studies have confirmed that PsyCap has positive effects on organizational commitment, job performance, job satisfaction, and well-being [24,27,28], but shows negative impacts on depression, burnout, and turnover in workplaces [12,14,20,21,22,29]. In addition, PsyCap plays a mediating role in the associations of occupational stress and work-family with depressive symptoms among Chinese physicians, university teachers, and nurses [12,14,22].

In China, with the rapid development of the society and economy, many kinds of occupational populations suffer from depression and its related symptoms. There is a high level of depressive symptoms in various Chinese occupational groups including nurses, physicians, male correctional officers, university teachers, and underground coal miners [11,12,13,14,15,22]. However, there is little research on depression or its related symptoms among bank employees in China and other countries [30]. The banking sector has been undergoing necessary changes with the development and reform of social economy. In general, there are a large number of customers in all kinds of banks in China, especially state-owned banks. Working in banks may be a profession that is particularly associated with high stress [30,31,32], as a result of intensive concentration, the need to hide negative emotions and answer various questions of customers. Because the state-owned banks have implemented enterprise management patterns that have intensified competition among the banks, front-line bank employees often have to suffer from high workload, and many of them showed high perceived stress and mental health problems [32,33,34]. However, there are no adequate rewards such as job promotion, stability, respect and income for them in the workplace [35]. Overall, the high effort of front-line bank employees is not met with adequate rewards, which indicates ERI. As a result, bank employees in failed social reciprocity are prone to depressive symptoms. Overcommitment refers to a personal cognitive pattern of coping with work tasks [9]. Individuals with overcommitment are supposed to work harder for a given task than non-overcommitted individuals, and they have the high risk of occurrence of depressive symptoms. Because front-line bank employees need to adhere to a strict schedule, they devote much time and energy to their work. Thus, many experience high levels of work-family conflict, which can contribute to depressive symptoms in the workplace. These adverse psychosocial working conditions tend to increase the susceptibility to depression among Chinese bank employees. Therefore, how to reduce the level of depression is not only the key to improving the mental health of bank employees but is also important to improve the service quality and benefits of banks. In addition, the mediating role of PsyCap in the association between occupational stress and job burnout had been found among bank employees in China. Investment in PsyCap seems to be crucial to reduce the depressive symptoms of bank employees.

In light of the above concerns, the objectives of this study were to explore, for Chinese bank employees, (1) the effects of occupational stress and work-family conflict on depressive symptoms; (2) the effect of PsyCap on depressive symptoms; and (3) whether the effects of occupational stress and work-family conflict on depressive symptoms are mediated by PsyCap.

## 2. Methods

### 2.1. Study Design and Sample

A cross-sectional survey was conducted in Liaoning province (population 43 million), China, from May to June in 2013. One city was randomly selected in each region (eastern, western, southern, northern and central) according to the geographic distributions of cities in Liaoning. Two banks were randomly selected from each selected city. About 50% of the front-line bank employees from each bank were randomly sampled. In total, 1546 bank employees were sampled, of whom 608 (39.3%) were males and 938 (60.7%) were females. A set of self-administered questionnaires was directly distributed to the bank employees after obtaining written informed consent. Complete responses were obtained from 1239 individuals (response rate: 80.1%), of whom 467 (37.7%) were males and 772 (62.3%) were females. The study was approved by the Committee on Human Experimentation of China Medical University (Ethical Project identification code: AF-SOP-07-1.0-01), and the study procedures were in accordance with ethical standards.

### 2.2. Measurement of Depressive Symptoms

The 20-item Chinese version of the Center for Epidemiologic Studies Depression (CES-D) Scale was adopted to measure the level of depressive symptoms in this study [36]. Each item contains four options that describe how often the respondents had each feeling in the past week, ranging from 0 “rarely or none of the time (less than 1 day)” to 3 “most or all of the time (5 to 7 days)”. The summed score ranged from 0 to 60, and a high score indicates a high level of depressive symptoms. The CES-D scale has been extensively validated and applied across Chinese occupational groups [11,12,13,14,15,22,25]. The Cronbach’s alpha for the CES-D scale was 0.834 for bank employees in the present study.

### 2.3. Measurement of Occupational Stress

The Chinese version of the ERI scale was used to assess occupational stress [37]. The ERI scale consists of three subscales and 23 items: extrinsic effort (6 items), reward (11 items), and overcommitment (6 items). For the extrinsic effort and reward measurement, participants initially express their attitude towards a working situation (“agree” or “disagree”), and then, they are asked to evaluate to what extent (from “not distressed” to “very distressed”) they usually feel distressed. For the overcommitment measurement, responses are scored from 1 “absolutely disagree” to 4 “absolutely agree”. High values of extrinsic effort and overcommitment indicate high level of occupational stress, whereas low value of reward indicates high level of occupational stress. The reliability and validity of the Chinese ERI scale have been widely confirmed across occupational groups [11,12,13,14,15]. In this study, the Cronbach’s alphas for the extrinsic effort, reward and overcommitment subscales were 0.889, 0.885, and 0.772, respectively.

### 2.4. Measurement of Work-Family Conflict

The level of work-family conflict was measured with the Chinese version of the WIF scale, which assesses the extent to which work demands interfere with family-related obligations [16]. The WIF scale consists of 9 items, and each item is scored on a 5-point Likert scale in which 1 indicates “never” and 5 indicates “always.” A high value indicates a high level of work-family conflict. Previous studies in various Chinese occupational populations have demonstrated good reliability and validity of the scale [20,21,22]. In the present study, the Cronbach’s alpha for the WIF scale was 0.949.

### 2.5. Measurement of PsyCap

The Chinese version of the 24-item Psychological Capital Questionnaire (PCQ) was used to measure the level of PsyCap [24]. The PCQ consists of four subscales (self-efficacy, hope, resilience, and optimism), and each subscale has six items. Each item is scored on a 6-point Likert scale with categories ranging from 1 “strongly disagree” to 6 “strongly agree”. The average score for the total scale was calculated in this study, and a high score indicated a high level of PsyCap. The Chinese PCQ has been extensively validated and applied across Chinese occupational groups [12,14,20,21,22,25,27]. For the PCQ scale, the Cronbach’s alpha was 0.925 in this study.

### 2.6. Demographic and Working Characteristics

Demographic and working factors included gender, age, marital status, education, weekly work time and monthly income. Marital status was categorized as “single” and “married/cohabitation”. Education was categorized as “junior college or lower”, “college”, and “graduate or higher”. Weekly work time was categorized as “≤40 h” and “>40 h”, according to the current work system of 8 h per day. Monthly income (RMB) was categorized as “≤3000 yuan”, “3001–5000 yuan”, and “>5000 yuan”.

### 2.7. Statistical Analysis

The probability-probability plot (PP plot) and Kolmogorov-Smirov (KS) tests were used to check the normal distribution of continuous variables before the data analyses were conducted. The group differences of depressive symptoms were examined using independent sample *t*-test or one way analysis of variance (ANOVA) with *post hoc* test (LSD). Pearson’s correlation analysis was executed to examine correlations among continuous variables. Hierarchical regression analysis was carried out to explore the effects of extrinsic effort, reward, overcommitment, work-family conflict, and PsyCap on depressive symptoms. In step 1, the demographic and working factors were put in the model as control variables. In step 2, extrinsic effort, reward, overcommitment, and work-family conflict were added. In step 3, PsyCap was added. According to Baron and Kenny’s procedure, the following are the conditions for determining mediation: (1) the independent variable (extrinsic effort/reward/overcommitment/work-family conflict) is significantly related to the dependent variable (depressive symptoms); (2) the independent variable is significantly related to the mediator (PsyCap); and (3) the mediator is significantly related to the dependent variable, with the effect of the independent variable on the dependent variable diminishing (partial mediator) or becoming statistically insignificant (full mediator) upon the addition of the mediator into the model. Then, the statistical significance of the mediating role of PsyCap (indicated by a*b product) was examined using Preacher and Hayes’ asymptotic and resampling strategies [38]. In these analyses, extrinsic effort, reward, overcommitment and work-family conflict were modeled as independent variables, depressive symptoms as dependent variable, PsyCap as mediator, and demographic and working characteristics as covariates. The bootstrap estimate was based on 5000 bootstrap samples. The bias-corrected and accelerated 95% confidence interval (BCa 95% CI) of the a*b product excluding 0 indicates a significant mediating role of PsyCap [12,39].

Before performing the regression analyses, all the continuous variables were centralized to account for differences in scale scores. Tolerance and variance inflation factor (VIF) were adopted to check for multi-collinearity. All analyses were conducted using SPSS 17.0 for Windows. A two-tailed *p* < 0.05 was considered to indicate statistical significance in all tests.

## 3. Results

### 3.1. Descriptive Statistics

The basic characteristics of the participants and mean scores of depressive symptoms are shown in Table 1. From all the participants, the mean score (standard deviation, SD) of depressive symptoms was 18.4 (7.6). The mean (SD) age was 33.9 (8.6) years and 62.3% were females. Of these participants, 64.8% were married or cohabiting, 71.7% possessed bachelor degrees, 40.8% had a monthly income level of ≤3000 yuan, and 67.2% had to work more than 40 h. In addition, there was a significant difference in the levels of depressive symptoms among age groups (*F* = 3.476, *p* < 0.05). LSD test showed that the level of depressive symptoms of bank employees in >40 years group was significantly higher than that of ≤30 years bank employees. In addition, there was a significant difference in the levels of depressive symptoms among monthly income groups (*F* = 6.540, *p* < 0.01). LSD test showed that the level of depressive symptoms of bank employees in monthly income ≤3000 yuan group was significantly higher than that of bank employees in 3001–5000 yuan group. Differences in gender, marital status, education and weekly working hours were not statistically significant.

**Table 1:** Participant characteristics, means and standard deviations (SDs) of depressive symptoms (*n* = 1239).

Variables	*n* (%)	Mean (SD)
Gender		
Male	467 (37.7%)	18.92 (7.70)
Female	772 (62.3%)	18.15 (7.47)
Age (years)		
≤30	560 (45.2%)	17.84 (7.63)
30–40	374 (30.2%)	18.74 (7.44)
>40	305 (24.6%)	19.16 (7.52) ^a,^*
Marital status		
Single	436 (35.2%)	17.94 (7.68)
Married/cohabitation	803 (64.8%)	18.71 (7.48)
Education		
Junior college or lower	126 (10.2%)	19.45 (6.94)
College	888 (71.7%)	18.52 (7.70)
Graduate or higher	225 (18.2%)	17.54 (7.28)
Weekly working time (hours)		
≤40	407 (32.8%)	17.93 (7.28)
>40	832 (67.2%)	18.68 (7.68)
Monthly income (yuan)		
≤3000	505 (40.8%)	19.25 (7.93) ^b,^**
3001–5000	597 (48.2%)	17.64 (7.15)
>5000	137 (11.0%)	18.89 (7.58)

Note: The investigation was performed from May to June in 2013. The group differences of depressive symptoms were examined using independent sample *t*-test or one way analysis of variance (ANOVA) with *post-hoc* test (LSD). SD: standard deviation. ^a^ significant difference between >40 years group and ≤30 years group; ^b^ significant difference between monthly income ≤3000 yuan group and 3001–5000 yuan group; * *p* < 0.05; ** *p* < 0.01 (two-tailed).

### 3.2. Correlations among Study Variables

The correlations among study variables are presented in Table 2. Age was negatively correlated with reward and PsyCap (*r* = −0.243, −0.075, *p* < 0.01), while it was positively correlated with depressive symptoms (*r* = 0.084, *p* < 0.01). Extrinsic effort, overcommitment and work-family conflict were positively correlated with depressive symptoms (*r* = 0.274, 0.197, 0.307, *p* < 0.01). Reward and PsyCap were negatively correlated with depressive symptoms (*r* = −0.227, −0.338, *p* < 0.01).

**Table 2:** Correlations among extrinsic effort, reward, overcommitment, work-family conflict, psychological capital (PsyCap) and depressive symptoms (*n* = 1239).

Variables	1	2	3	4	5	6
1. Age	1					
2. Extrinsic effort	0.028	1				
3. Reward	−0.243 **	−0.223 **	1			
4. Overcommitment	0.019	0.367 **	−0.144 **	1		
5. Work-family conflict	−0.033	0.328 **	−0.068 *	0.259 **	1	
6. PsyCap	−0.075 **	−0.254 **	0.235 **	−0.125 **	−0.120 **	1
7. Depressive symptoms	0.084 **	0.274 **	−0.227 **	0.197 **	0.307 **	−0.338 **

Note: The investigation was performed from May to June in 2013. Pearson’s correlation analysis was executed to examine correlations among continuous variables. PsyCap: psychological capital. * *p* < 0.05; ** *p* < 0.01 (two-tailed).

### 3.3. Associations of Occupational Stress, Work-Family Conflict and PsyCap with Depressive Symptoms

As shown in Table 3, the tolerance values of all independent variables were more than 0.75 (tolerance = 0.752–0.970), and the VIFs of all independent variables were less than 1.33 (VIF = 1.031–1.329). These values suggested that multi-collinearity was not a problem in the estimates presented in our study. Gender and age were not significantly associated with depressive symptoms when the three dimensions of occupational stress, work-family conflict and PsyCap were added to the equation successively in step 3. In step 2, extrinsic effort, overcommitment and work-family conflict were positively associated with depressive symptoms, and reward was negatively associated with depressive symptoms, explaining 15.4% of the variance in depressive symptoms. In step 3, the effect of PsyCap on depressive symptoms was significantly negative, accounting for an additional 5.4% of the variance. The absolute standardized partial regression coefficients of extrinsic effort, reward, overcommitment and work-family conflict diminished when PsyCap was added (extrinsic effort: from β = 0.131 to β = 0.085, reward: from β = 0.066 to β = 0.062, overcommitment: from β = 0.159 to β = 0.112, work-family conflict: from β = 0.239 to β = 0.229). These results implied that PsyCap could probably become a mediator in the associations of occupational stress and work-family conflict with depressive symptoms.

### 3.4. PsyCap’s Mediating Roles in the Associations of Occupational Stress and Work-Family Conflict with Depressive Symptoms

The associations of occupational stress and work-family conflict with PsyCap (a), a*b products (b, the associations of PsyCap with depressive symptoms after controlling for other independent variables) and BCa 95% CI for these products are presented in Table 4. Extrinsic effort and reward were significantly associated with PsyCap (a_â_ = −0.186, 0.188, *p* < 0.01). Because PsyCap was significantly and negatively associated with depressive symptoms, the significant mediating roles of PsyCap in the associations of extrinsic effort (a*b = 0.046, BCa 95% CI: 0.029, 0.066) and reward (a*b = −0.047, BCa 95% CI: −0.065, −0.030) with depressive symptoms were revealed. The proportions of the total effects of extrinsic effort and reward on depressive symptoms mediated by PsyCap were 35.1% and 29.6%, respectively.

## 4. Discussion

This is the first study to explore the effects of occupational stress, work-family conflict and PsyCap on depressive symptoms, and further examined the mediating roles of PsyCap among Chinese bank employees. The subjects of this study were selected from the state-owned banks located in different regions of Liaoning province, with a high response rate of 80.1%. These study subjects were representative, enhancing the generalizability of the study conclusions.

**Table 3:** Hierarchical linear regression analysis results with depressive symptoms as criterion variable (*n* = 1239).

Variables	Depressive Symptoms								
	Step 1			Step 2			Step 3		
	â	Tolerance	VIF	â	Tolerance	VIF	â	Tolerance	VIF
Gender	−0.037	0.970	1.031	−0.031	0.955	1.047	−0.043	0.953	1.050
Age	0.090 **	0.961	1.041	0.057 *	0.911	1.098	0.046	0.909	1.100
Middle *vs.* low income	−0.118 **	0.873	1.145	−0.110 **	0.869	1.151	−0.091 **	0.864	1.158
High *vs.* low income	−0.029	0.869	1.150	−0.054	0.865	1.156	−0.044	0.864	1.158
Extrinsic effort				0.131 **	0.775	1.290	0.085 **	0.752	1.329
Reward				−0.159 **	0.876	1.141	−0.112 **	0.847	1.181
Overcommitment				0.066 *	0.839	1.193	0.062 *	0.838	1.193
Work-family conflict				0.239 **	0.866	1.154	0.229 **	0.865	1.156
PsyCap							−0.247 **	0.892	1.121
*F*	6.537 **			32.545 **			40.573 **		
*R*^2^	0.021			0.175			0.229		
∆*R*^2^	0.021 **			0.154 **			0.054 **		

Note: The investigation was performed from May to June in 2013. Hierarchical regression analysis was carried out to explore the effects of extrinsic effort, reward, overcommitment, work-family conflict and PsyCap on depressive symptoms. Gender: female *vs.* male; Low income: ≤3000 yuan; Middle income: 3001–5000 yuan; High income: >5000 yuan; VIF: variance inflation factor; PsyCap: psychological capital. The values of tolerance and VIF suggested that multi-collinearity could be accepted in these regression models. * *p* < 0.05; ** *p* < 0.01 (two-tailed).

**Table 4:** Asymptotic and resampling regression analysis results with PsyCap as mediator (*n* = 1239).

Independent Variables	a (â)	a*b (BCa 95% CI)
Extrinsic effort	−0.186 **	0.046 * (0.029, 0.066)
Reward	0.189 **	−0.047 * (−0.065, −0.030)
Overcommitment	−0.018	0.004 (−0.011, 0.019)
Work-family conflict	−0.042	0.011 (−0.005, 0.028)

Note: The investigation was performed from May to June in 2013. The statistical significance of the mediating role of PsyCap (indicated by a*b product) was examined using Preacher and Hayes’ asymptotic and resampling strategies. PsyCap: psychological capital, a: associations of occupational stress and work-family conflict with PsyCap; b: association of PsyCap with depressive symptoms after controlling for other independent variables; a*b: Product of a and b. BCa 95% CI: bias-corrected and accelerated 95% confidence interval. Gender, age, middle (3001–5000 yuan) *vs.* low (≤3000 yuan) income, and high (>5000 yuan) *vs.* low income were covariates. * *p* < 0.05; ** *p*< 0.01 (two-tailed).

Although the average score of depressive symptoms of Chinese bank employees was lower than that of physicians and underground coal miners [12,22], it was higher than that of non-frontline correctional officers in China [39], and was similar to that of Chinese university teachers, nurses, and frontline correctional officers [14,22,39]. Unfortunately, the comparison on the level of depressive symptoms across studies and countries could not be carried out, because there is no study on the symptoms of depression of bank employees using the same measurement tool in China and other countries to our best knowledge. As a whole, our results supported the assertion that Chinese bank employees suffer from high level of depressive symptoms. Therefore, urgent efforts should be made to prevent and reduce these symptoms among Chinese bank employees.

In view of the risk factors of depressive symptoms, occupational stress and work-family conflict were associated with depressive symptoms among Chinese bank employees. This finding was consistent with previous studies of other occupational groups from different countries [11,12,13,14,15,19,22]. Extrinsic effort, overcommitment and work-family conflict had positive associations with depressive symptoms, while reward had a negative effect on depressive symptoms in this study. In Brazil, Valente *et al.* reported that high effort/low reward, overcommitment and ERI were associated with depressive symptoms in bank employees [30]. On one hand, bank employees, engaged in a service profession, are rarely understood and praised by customers, and need to accomplish certain performance goals [35]. They do not have enough time to relieve competitive pressure from being laid off and policy reform. On the other hand, bank employees who experience more work-family conflicts devote more time and energy to work at the expense of interfering with family-related obligations, which makes them physically and mentally exhausted [40]. In the bank workplace, many employees might become burned out, unable to cope with so much pressure, and, thus, they fall into a depressive state. Our results suggested that there is a great need for bank administrators to develop and implement effective measures to alleviate employees’ depressive symptoms and improve their mental health. Bank administrators should try to reduce workload and working hours and to provide appropriate awards based on employees’ abilities and performance.

As an important concept of organizational behavior, PsyCap has been considered as a positive resource to combat the negative outcomes of destructive emotions, job stress, burnout, and work-family conflict [12,14,20,21,22,32,39]. This study showed a significantly negative association of PsyCap with depressive symptoms among Chinese bank employees. In addition, occupational stressors from ERI (extrinsic effort and reward) were significantly associated with PsyCap, which motivated us to explore their indirect effects in predicting depressive symptoms through PsyCap. In fact, PsyCap partially mediated the associations of extrinsic effort and reward with depressive symptoms, which indicated that PsyCap could be a positive resource to alleviate the depressive symptoms of Chinese bank employees, and reduce the adverse effects of occupational stress on mental health. Bank employees who experienced more extrinsic effort or less reward would be more likely to possess low levels of PsyCap which would increase the possibilities and severity of depressive symptoms.

Therefore, it is important for bank administrators to note that strategies to enhance employees’ PsyCap seem to be crucial to improve depressive symptoms and promote mental health of bank employees in China. As we mentioned, PsyCap is defined as state-like and is open to development [23,24]. Therefore, developing employees’ self-efficacy, hope, resilience and optimism is critical in the bank workplaces. This implied that Chinese bank employees, with adequate level of PsyCap, are less likely to feel tired and tend to alleviate depressive symptoms. Based on the findings from some previous studies on the development PsyCap, positive thinking habits and rational cognitive ability should be cultivated among employees at the individual level. Besides, employees should set appropriate and challenging job goals to boost their confidence [24,41]. At the organizational level, organizational support and congratulation should be strengthened and make individuals feel a friendly and harmonious atmosphere [38,42,43]. With the enhancement of Psycap, bank employees’ attitude to work and life will be more optimistic.

In spite of these merits, several limitations of the present study have to be mentioned. First, our study was a cross-sectional design, and thus we are unable to assess the causal relations among study variables. However, our study hypotheses were built on solid theoretical and research foundations. The results from the cross-sectional study need to be confirmed with a prospective cohort study. Second, the study population comprised staff from state-owned banks in urban areas only. The employees from private or municipal banks and those working in the countryside were not included, which could limit the representativeness of our study sample and the generalizability of the results. Third, some other factors associated with depressive symptoms in organizational and social environments, such as lack of organizational or social supports, transactional leadership and organizational climate, as well as individual stressors, such as role conflict, coping style and personality should be investigated in further studies. In addition, possible confounders such as life events, job rank and organizational efficiency should be considered in further studies.

## 5. Conclusions

There is a high level of depressive symptoms among Chinese bank employees. Extrinsic effort, overcommitment and work-family conflict were positively associated with depressive symptoms, whereas reward and PsyCap were negatively associated with depressive symptoms. PsyCap partially mediated the effects of extrinsic effort and reward on depressive symptoms. Investing in PsyCap may provide new approaches to improving mental health among Chinese bank employees.
